# Reconstruction of diaminopimelic acid biosynthesis allows characterisation of *Mycobacterium tuberculosis N*-succinyl-L,L-diaminopimelic acid desuccinylase

**DOI:** 10.1038/srep23191

**Published:** 2016-03-15

**Authors:** Veeraraghavan Usha, Adrian J. Lloyd, David I. Roper, Christopher G. Dowson, Guennadi Kozlov, Kalle Gehring, Smita Chauhan, Hasan T. Imam, Claudia A. Blindauer, Gurdyal S. Besra

**Affiliations:** 1 School of Biosciences, University of Birmingham, Edgbaston, Birmingham B15 2TT, UK; 2 School of Life Sciences, University of Warwick, Coventry, CV4 7AL, UK; 3 Department of Biochemistry, McGill University, Montreal, Quebec, H3G 1Y6, Canada; 4 Department of Chemistry, University of Warwick, Coventry, CV4 7AL,UK

## Abstract

With the increased incidence of tuberculosis (TB) caused by *Mycobacterium tuberculosis* there is an urgent need for new and better anti-tubercular drugs. N-succinyl-**L**,**L**-diaminopimelic acid desuccinylase (DapE) is a key enzyme in the succinylase pathway for the biosynthesis of meso-diaminopimelic acid (meso-DAP) and **L**-lysine. DapE is a zinc containing metallohydrolase which hydrolyses N-succinyl **L**,**L** diaminopimelic acid (**L**,**L**-NSDAP) to **L**,**L**-diaminopimelic acid (**L**,**L**-DAP) and succinate. *M. tuberculosis* DapE (MtDapE) was cloned, over-expressed and purified as an N-terminal hexahistidine ((His)_6_) tagged fusion containing one zinc ion per DapE monomer. We redesigned the DAP synthetic pathway to generate **L**,**L**-NSDAP and other **L**,**L**-NSDAP derivatives and have characterised MtDapE with these substrates. In contrast to its other Gram negative homologues, the MtDapE was insensitive to inhibition by **L**-captopril which we show is consistent with novel mycobacterial alterations in the binding site of this drug.

TB is a major cause of mortality. In 2011, there were 12 million cases of TB of which an estimated 1.4 million were fatal^1^. The spread of multi, extremely and totally drug resistant TB has necessitated the identification and characterisation of new drug targets to treat TB^2^.

The DAP biosynthetic pathway operates only in bacteria and plants. It synthesises *meso*-DAP (or **D**,**L**-DAP), the precursor of **L**-lysine^3^,^4^. *M. tuberculosis* incorporates DAP into the stem peptide of its peptidoglycan^5^,^6^. Therefore, *meso-*DAP synthesis is an attractive antimicrobial target because it targets both peptidoglycan and protein synthesis. Hence, a DapE inhibitor would neither be toxic or undermined by the presence of any exogenous source of the diamino acid in the human host^7^.

There are three pathways of DAP synthesis^3^,^4^,^7^ and all three involve synthesis of **L**-2,3,4,5-tetrahydrodipicolinic acid (**L**-THDP). In the succinylase pathway, **L**-THDP is acylated usually with succinyl-CoA by DapD (acyl-CoA:THDP *N*-acyl transferase), to form *N*-succinyl-2-amino-6-ketopimelic acid (NS-AKP), which is transaminated with **L**-glutamate by DapC (*N*-acyl-DAP aminotransferase) or its homologue ArgD (acetylornithine/succinyl DAP aminotransferase) to furnish **L**,**L**-NSDAP. This is hydrolysed to succinate and **L**,**L**-DAP by DapE (E.C. 3.5.1.18) which in *M. tuberculosis* (MtDapE) is encoded by *dapE* (Rv1202). **L**,**L**-DAP is epimerized to *meso*-DAP by DAP epimerase (DapF). In the dehydrogenase pathway, **L**-THDP is directly reduced to *meso*-DAP by NADP^+^-linked *meso*-DAP dehydrogenase^3^,^4^,^7^. The acetylase pathway consists of three reaction steps prior to formation of **L**,**L**-DAP which is converted to *meso*-DAP by DapF^8^.

DapE typically possesses two catalytic metal binding sites^9^,^10^: one of high affinity which is essential and is always occupied by zinc and the other of low affinity which is not essential for catalysis and loses metal upon extensive dialysis^11^,^12^. Evidence of a dinuclear zinc active site in the *Haemophilus influenza* DapE (HiDapE) and the *Neisseria meningitidis* DapE (NmDapE) was obtained *via* extended X-ray absorption fine spectra (EXAFS)^13^ and X-ray crystallography^9^,^10^. The presence of zinc in the active site of DapE has been exploited as a target for thiol-containing inhibitors, such as **L**-captopril^10^,^14^,^15^.

The crystal structures of both mono and dinuclear zinc forms of HiDapE have been solved^9^. MtDapE has also recently been crystallised^16^. Inspection of the sequence of the structurally characterised HiDapE and that of other DapE sequences reveals strict conservation of all metal ligand and substrate binding residues^12^,^17^. Two highly conserved histidines that are present in the active site act as zinc ligands^12^,^17^.

Deletion of the gene encoding DapE is lethal to *Helicobacter pylori* and *M.smegmatis*^18^,^19^ but can be complemented by DAP-supplementation^18^. Consequently there has been interest in this protein as a potential antimicrobial target^14^. However, an impediment to the facile characterization of DapE, and its exploitation as a drug target is the unavailability of its substrate. Therefore we devised a method to synthesise **L**,**L**-NSDAP and analogues thereof using a novel *in vitro* pathway constructed from the two natural DAP synthetic pathways (Fig. 1a). To achieve this, we over-expressed and purified MtDapE, *Corynebacterium glutamicum* DAP dehydrogenase (Cg*meso*-DAP dehydrogenase), *Bacillus anthracis* DapF (BaDapF), *Escherichia coli* ArgD (EcArgD) and *E. coli* DapD (EcDapD).

## Results & Discussion

### Overexpression and purification of MtDapE, Cg*meso*-DAP dehydrogenase, BaDapF, EcArgD and EcDapD

MtDapE was obtained in the soluble fraction which was purified by a single step Ni^2+^ Sepharose affinity chromatography. The purified MtDapE fractions were electrophoretically homogeneous (Supplementary Fig. S1a). The final yield was 2–3 mg of pure MtDapE per litre of culture. Activity of purified recombinant MtDapE was stable at −80 °C for about 3 weeks. Cg*meso*-DAP dehydrogenase and BaDapF were similarly purified to electrophoretic homogeneity as judged by SDS-PAGE (Supplementary Fig. S1b and S1c). EcArgD and EcDapD were also purified to apparent homogeneity (Supplementary Fig. S1d and S1e) and yields of about 10 mg of EcArgD and 15 mg of EcDapD were obtained per litre of culture.

### Metal content and dependence of MtDapE

DapE homologues from other organisms require a tightly bound zinc ion for catalysis^9^,^10^,^11^,^12^,^13^. To determine whether MtDapE was similar in this regard, we removed zinc from pure MtDapE by dialysis against EDTA and 1, 10-phenanthroline. This resulted in complete loss of MtDapE activity. We then analysed the untreated and metal chelator treated MtDapE by inductively coupled plasma optical emission spectroscopy (Methods section). The treated enzyme contained no detectable Zn^2+^. In contrast, untreated MtDapE contained 0.8 ± 0.1 Zn^2+^ per monomer and was enzymatically active.

DapE proteins when fully substituted contain two zinc/monomer^9^,^10^,^20^. However, there is little difference in catalysis incurred on the loss of a single zinc atom from the active site^10^,^11^,^12^,^15^. This has led to postulation of a catalytic mechanism, wherein histidine 349 of HiDapE (H330 in MtDapE) takes on the role of the second zinc ion in correctly orientating **L**,**L**-NSDAP within the active site^9^. Our observation of the activity of a singly zinc-substituted MtDapE is consistent with the operation of a similar His-dependent mechanism in this mycobacterial enzyme.

### Spectrophotometric assay for an enzymatic synthesis of L,L-NSDAP, L,L-NGDAP and other DAP derivatives

**Figure 1:**
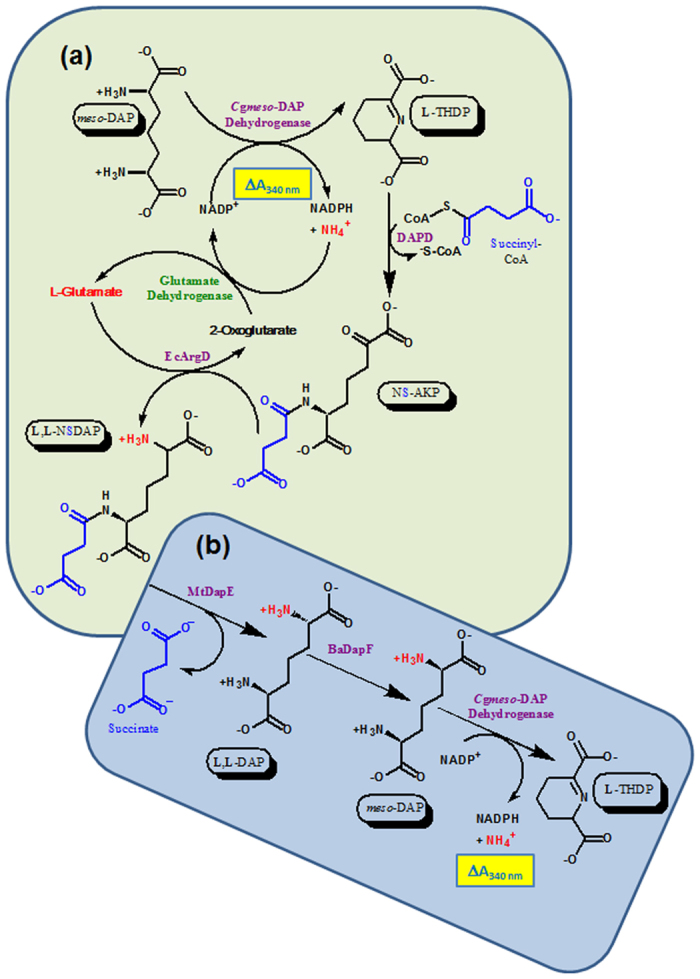
Synthesis and detection of L,L-NSDAP. **(a)** Synthesis: *meso*-DAP is oxidised with NADP^+^ to **L**-THDP by Cg*meso*-DAP dehydrogenase, yielding an increase in absorbance at 340 nm. **L**-THDP is succinylated with succinyl-CoA (or acylated by an alternative acyl-CoA) by EcDapD to yield NS-AKP which is amidated with glutamate to form **L**,**L**-NSDAP or its acyl derivative by EcArgD. Reaction condition 1 (see text) is performed in this way. Reaction condition 2 (see text) recycles the NADPH and ammonia produced by Cg*meso*-DAP dehydrogenase with glutamate dehydrogenase (green broken line). **(b)** Detection: **L**,**L**-NSDAP is cleaved by MtDapE to succinate and **L**,**L**-DAP which is epimerised by BaDapF and reduced by Cg*meso*-DAP dehydrogenase with a concomitant increase in absorbance at 340 nm.

In order to characterise MtDapE, it was necessary to devise a convenient method for the synthesis of its substrate. We anticipated we could reverse the DAP dehydrogenase step, generating **L**-THDP from *meso-*DAP and NADP^+^ with *E. coli* DapD, succinyl-CoA (or other acyl-CoAs), *E. coli* DapC or its orthologue *E. coli* ArgD and glutamate, which would generate **L**,**L**-NSDAP, or acyl analogues thereof (Fig. 1a). Furthermore, we reasoned that we could follow this process by the addition of MtDapE and BaDapF allowing regeneration of *meso-*DAP which could be detected by the Cg*meso*-DAP dehydrogenase originally present for the initial formation of **L**-THDP (Fig. 1b).

**Figure 2:**
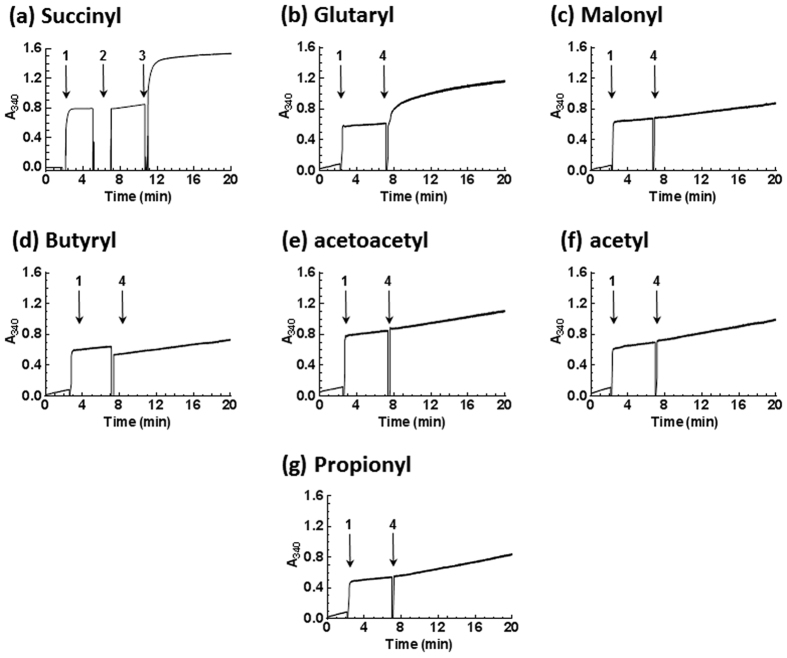
Spectrophotometric assay of the enzymatic synthesis of N-acyl L,L DAP. All assays contained 20 mMTricine buffer pH 8.0, 10 mM MgCl_2_, 10 mM NADP^+^, 1.99 μM Cg*meso*-DAP dehydrogenase, 8.03 μM DapD, 10 mM **L**-glutamate, 8.97 μM EcArgD, 2 mM PLP and 20 mM DTT at 0 time. The identity of the acyl group of the CoA thiol ester substrate is as indicated. **(a)** Succinyl-CoA: At **1**, 0.1 mM *meso*-DAP was added. Once reduction of *meso*-DAP to **L**-THDP was complete, 0.1 mM succinyl CoA and 0.134 μM MtDapE was added at **2**. Finally, 22.8 μM BaDapF was added at **3**; **(b)** Glutaryl-CoA, **(c)** Malonyl-CoA, **(d)** Butyryl-CoA, **(e)** Acetoacetyl-CoA, **(f)** Acetyl-CoA and **(g)** Propionyl-CoA: Included in addition to the components present at 0 time in Panel **2(a)** was 0.1 mM acyl-CoA as specified and 0.134 μM MtDapE. The absorbance at 340 nm was monitored at 37 °C. At **1**, 0.1 mM *meso*-DAP was added. At **4**, 22.8 μM BaDapF was added.

In these initial spectrophotometric experiments, we maintained the succinyl-CoA substrate of EcDapD at a concentration equal to that of *meso-*DAP to ensure a single turnover of the DAP added (detailed Fig. 2 legend). We were then able to monitor the synthesis of **L**,**L-**NSDAP as a function of NADPH synthesis with a concomitant increase in absorbance at 340 nm (Fig. 2a).

Consistent with this pathway, a jump in absorbance at 340 nm occurred on the addition of *meso-*DAP consistent with the complete oxidation of the latter. No further increase in absorbance was detected on addition of EcDapD or EcArgD until BaDapF was added when there was an additional increase in absorbance equal to that on the addition of *meso-*DAP (Fig. 2a). This second increase in absorbance was dependent on EcArgD, EcDapD, BaDapF, succinyl-CoA and *meso-*DAP and was consistent with the formation of **L**,**L**-NSDAP and its consumption by DapE.

To test the flexibility of this system with respect to generation of acyl-**L**,**L**-DAP analogues of **L**,**L**-NSDAP, we repeated the above pathway reconstruction assays replacing succinyl-CoA with glutaryl, malonyl, butyryl, acetoacetyl, acetyl, and propionyl-CoA thiol esters (Fig. 2b–g). Glutaryl-CoA supported synthesis of a MtDapE active substrate almost as efficiently as succinyl-CoA (Fig. 2b). Far smaller or negligible quantities of product were generated with the other acyl-CoA thiol esters within the time frame of the experiment (Fig. 2c–g).

We therefore tested the possibility that malonyl, butyryl, acetoacetyl, acetyl or propionyl-CoA thiol esters could not support the EcDapD activity efficiently enough to generate detectable quantities of acyl-**L**,**L**-DAP analogues over duration of the assay. Therefore, we assayed EcDapD and **L**-THDP dependent acyl-CoA deacylation *via* reduction of 5,5′dithio*bis*(2-nitrobenzoic acid) by CoA thiol to *para*-thionitrobenzoate at 412 nm^21^ (Methods section; Supplementary Fig. S2a,S2b).

EcDapD assays showed that the best acyl-CoA substrate was succinyl-CoA (100%), followed by glutaryl-CoA (4.4%), acetoacetyl-CoA (0.042%), acetyl-CoA (0.042%), malonyl-CoA (0.022%) and butyryl-CoA (0.0018%). No EcDapD activity was supported by propionyl-CoA over the duration of the assay. The data suggested that insignificant generation of these acyl-**L**,**L**-DAP products may well be a consequence of the substrate specificity of EcDapD.

These results were consistent with the crystal structure of the MtDapD: succinyl-CoA complex where a correctly sited carboxyl of the succinyl-CoA substrate is required to form electrostatic and hydrogen bonding interactions with MtDapD^21^.

### Enzymatic synthesis, purification and characterisation of L,L-NSDAP, L,L-NGDAP and other DAP derivatives

To synthesise MtDapE substrates preparatively, we reconfigured the spectrophotometric assay of **L**,**L**-acyl-DAP synthesis as a single pot method using EcArgD, EcDapD and Cg*meso*-DAP dehydrogenase (Methods section; Fig. 1a, reaction condition 1). Additionally, in an alternate reaction scheme (Fig. 1a, green dotted line) NADPH generated by Cg*meso*-DAP dehydrogenase could be continuously reconverted to the starting NADP^+^ by **L**-glutamate dehydrogenase which consumed the 2-oxoglutarate generated by EcArgD and the NADPH and ammonia generated by Cg*meso*-DAP dehydrogenase (Methods section; reaction condition 2). This reduced the amount of nicotinamide cofactor used in the synthesis, making purification of the desired products easier. Similar strategies have been employed for synthesis of peptidoglycan precursors^22^.

**Figure 3:**
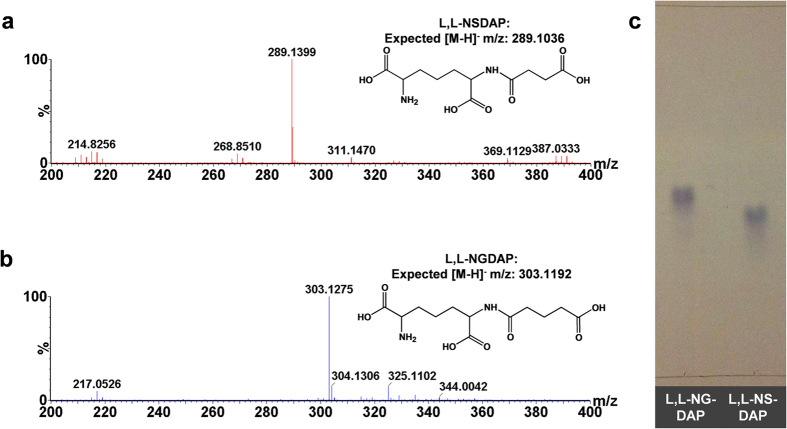
Characterization of L,L-NSDAP and L,L-NGDAP synthesised using Cg*meso*DAP dehydrogenase, EcArgD and EcDapD. **(a)** The electrospray negative ion mass spectrum of **L**,**L**-NSDAP. Expected [M-H] = 289.1036. **(b)** The electrospray negative ion mass spectra of **L**,**L**-NGDAP. Expected [M-H] = 303.1192. **(c)** Cellulose TLC assessment of purity of **L**,**L**-NGDAP (Lane 1) and **L**,**L**-NSDAP (Lane 2). The samples were spotted and developed in methanol: water: concentrated hydrochloric acid: pyridine (90: 7.5: 2.5: 10), and detected by ninhydrin staining.

We recovered 4.42 μmols or 3.47 μmols **L**,**L**-NSDAP in 27.6 and 21.6% overall yield from reaction conditions 1 and 2, respectively. The identity of **L**,**L-**NSDAP was confirmed by negative ion nanospray-MS analysis where the respective expected and observed values for the singly charged **L**,**L-**NSDAP [M-H]^−^ ion were 289.1036; and 289.1399 m/z (Fig. 3a). As confirmed by TLC, **L**,**L-**NSDAP obtained from reaction condition 1 was highly pure (Fig. 3c). No degradation of **L**,**L**-NSDAP was observed even after two years of storage at −80 °C.

The ability to generate the MtDapE substrate lent itself to the synthesis of a number of acyl-analogues of **L,L-**NSDAP that we could use to probe the substrate specificity of MtDapE. We therefore pursued the synthesis of a number of **L**,**L**-acyl-DAP derivatives by varying the coenzyme A acyl donor utilised by DapD. However, cognisant of the negligible yields of acyl-**L**,**L**-DAP species afforded by most of the acyl-CoA species utilised in Fig. 2, we extended the incubation time to overnight to maximise the possibility of synthesising products from even the acyl-CoA donors that were least reactive in the spectrophotometric assay of enzymatic synthesis of N-**L**,**L**-acyl-DAP derivatives (Fig. 2d,f).

Following purification of the products of the attempted syntheses of the various N-**L**,**L**-acyl-DAP derivatives, using DapE assays to follow elution of N-**L**,**L**-acyl-DAP species, we were able to confirm the synthesis of **L**,**L**-N-glutaryl-DAP (**L**,**L**-NG-DAP) enzymatically in 30.1% yield. TLC analysis of this species suggested it was essentially homogeneous (Fig. 3c). Negative ion nanospray-MS analysis (Fig. 3b) where the respective expected and observed m/z values for the singly charged **L**,**L-**NGDAP [M-H]^−^ ion were 303.1192 and 303.1275 m/z confirmed the synthesis of this species.

### Synthesis of L,L-N-malonyl, butyryl, acetoacetyl, acetyl and propionyl–DAP

Using absorbance at 215 nm to follow purification of N-**L**,**L**-acyl-DAP species (Methods section), we purified the products arising from syntheses designed to generate malonyl, acetoacetyl and butyryl-**L**,**L**-DAP. Negative ion nanospray-MS analysis indicated that although these compounds had been synthesised, they were characterised by very low ion intensities compared to contaminating ions in the corresponding mass spectra. Here, the expected/observed m/z values for **L**,**L-**N-malonyl-DAP and **L**,**L-**N-butyryl-DAP, respectively, were [M-H]^−^: 259.12939/259.0853 and 275.08792/275.08546; and for [M+Na-H]^−^ were 281.1113/281.1131 and 297.0546/297.0120, respectively. The corresponding expected/observed m/z value expected for **L**,**L-**N-acetoacetyl-DAP [M+Na-H]^−^ was 295.096/295.1061. No ions could be detected confirming the synthesis of acetyl- or propionyl-**L**,**L**-DAP.

Apart from **L**,**L-**NSDAP and **L**,**L-**NGDAP, of the acyl-DAP species whose synthesis could be confirmed by mass spectrometry, only the synthesis designed to generate acetoacetyl-**L**,**L**-DAP produced sufficient material to quantitate gravimetrically (5.56 μmols, equating to an overall yield of 22.2%). However, here, the lack of purity of the acetoacetyl-**L**,**L**-DAP product suggested by its mass spectral analysis indicated the true yield of this DAP-derivative was considerably less than that suggested by weight. Clearly, the current methodology did not lend itself to the generation of significant quantities of the remaining acyl-**L**,**L**-DAP species whose synthesis was attempted. This was in large part due to the restrictive substrate specificity of EcDapD.

Although synthesis of the **L**,**L-**N-malonyl, butyryl, acetoacetyl, acetyl and pyopionyl–DAP species was attempted, the yields were low enough to preclude their characterization as DapE substrates. Therefore, as a preparative technique our enzymatic approach lacked the flexibility of chemical syntheses that have generated **L**,**L-**N-acetyl-DAP, **L**,**L-**N-butyryl-DAP, and **L**,**L**-NGDAP^23^,^24^. It does however suggest that this method could generate possibly larger analogues of **L**,**L-**NSDAP such as **L**,**L**-NG-DAP and **L**,**L-**N-pimeloyl-DAP, as well as a range of NMR-active or radio labelled **L**,**L-**NSDAP species in laboratories unequipped for stereo-chemically controlled organic synthesis required to access DAP analogues^4^.

### Kinetic characterisation of MtDapE

**Figure 4:**
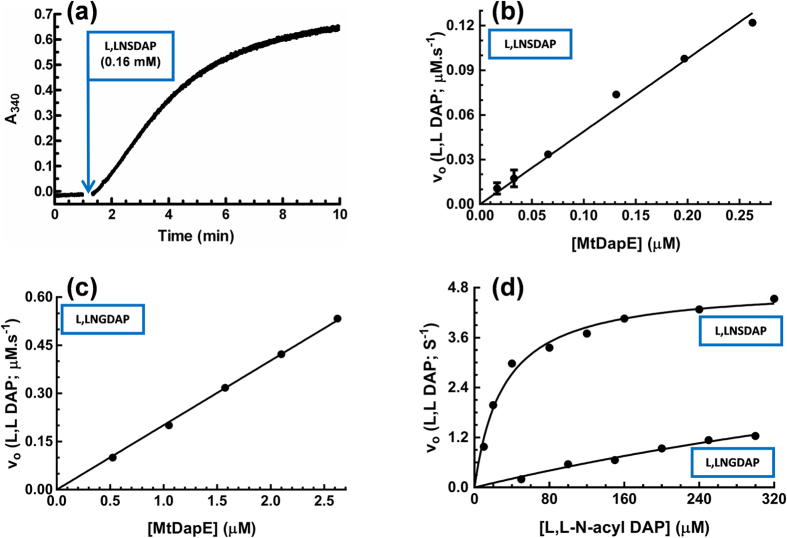
Time course and dependence of catalysis of L,L N-acyl DAP hydrolysis on MtDapE and L,L N-acyl DAP concentration. All data points are means of duplicates that differ by no more than 10%. **(a)** Time course of **L**,**L**-NSDAP hydrolysis by MtDapE. Conditions as in the text except the [**L**,**L**-NSDAP] was 0.16 mM.. MtDapE activity was initiated at 1 minute by addition of substrate; **(b)** Dependence of initial velocity of **L**,**L**-NSDAP hydrolysis on MtDapE concentration. **L**,**L**-NSDAP was at 31 μM; **(c)** Dependence of initial velocity of **L**,**L**-NGDAP hydrolysis on MtDapE concentration. **L**,**L**-NGDAP was at 72.46 μM; **(d)** Dependence of initial velocity of MtDapE-catalysed **L**,**L**-N-acyl-DAP hydrolysis on **L**,**L**-N-acyl-DAP concentration. For assays in the presence of **L**,**L**-NSDAP and **L**,**L**-NGDAP, MtDapE was at 0.134 μM and 1.05 μM respectively. Data were fitted to the Michaelis Menten equation by non-linear regression with GraphPad™ Prizm 4.0.

Having achieved a viable synthesis of **L**,**L**-NSDAP, we then kinetically characterised MtDapE where its production of **L**,**L**-DAP from **L**,**L**-NSDAP could be monitored at 340 nm by coupling the enzyme to BaDapF and Cg*meso*-DAP dehydrogenase catalysed reduction of NADP^+^ (Methods section). **L**,**L**-DAP production was dependent upon MtDapE and **L**,**L**-NSDAP (Fig. 4). Progress curves (Fig. 4a) of MtDapE showed a steady state preceded by a short lag of 10 to 20 seconds. The end point of the assay indicated almost complete consumption of the **L**,**L**-NSDAP in the assay. The relationship between DapE concentration and rate was strictly linear (Fig. 4b), indicating the assay quantitatively reported DapE activity.

The dependence of MtDapE activity on **L**,**L**-NSDAP concentration was hyperbolic and could be fitted to the Michaelis Menten equation (Fig. 4d) where the MtDapE *K*_m_, k_cat_ and k_cat_/*K*_m_ ratio for **L**,**L**-NSDAP were 31.09 ± 3.71 μM, 4.85 ± 0.15 s^−1^ and 0.156μM.s respectively. This latter value is similar to that of *E. coli* DapE enzyme^25^ suggesting that these DapE homologues had similar catalytic efficiencies.

The temperature and pH optima for DapE catalysis were determined. The temperature optimum of the reaction at pH 8.0 was between 37 to 42 °C (Supplementary Fig. S3a). The relationship between MtDapE activity and pH was bell-shaped with a pH optimum of 7.5 (Supplementary Fig. S3b). To ensure that this reflected MtDapE activity, the experiment was repeated at four-fold higher Cg*meso*-DAP dehydrogenase or BaDapF concentrations (7.96 μM and 91.2 μM respectively), with similar results (Supplementary Fig. S3c and S3d). This suggested that the pH profile in Supplementary Fig. S3b reported the impact of pH on MtDapE activity.

The pH profile of MtDapE resembled that of HiDapE^12^. The half maximal values of the ascending limb of the pH profile for MtDapE was 6.7 (Supplementary Fig. S3b). This value could represent dissociation of the zinc-activated water molecule central to the DapE mechanism^12^ or the dissociation of H352 in MtDapE whose corresponding residue in the mono-zinc substituted HiDapE crystal structure (H349) has been implicated in orientating **L**,**L**-NSDAP within the active site^9^. The half maximal value of the descending limb of the pH profile for MtDapE was 8.2 (Supplementary Fig. S3b). This could relate to dissociation of MtDapE amino acids or the free amino group of **L**,**L**-NSDAP or both^12^.

In order to probe the substrate structure-activity relationship of MtDapE, we sought to synthesise and test other acyl-**L**,**L**-DAP species. In this regard, MtDapE utilised **L**,**L**-NG-DAP as a substrate. We established that the assay utilising **L**,**L**-NG-DAP was linearly dependent on MtDapE protein (Fig. 4c) and found MtDapE to have a *K*_m_ for **L**,**L**-NGDAP of 1024 ± 645μM and a k_cat_ of 5.60 ± 2.88 s^−1^ where the k_cat_/*K*_m_ ratio was 0.00547μM.s (Fig. 4d). The imprecision of these constants relates to the very high *K*_*m*_ for **L**,**L**-NGDAP. Nevertheless on comparison of k_cat_/*K*_m_ ratios, **L**,**L**-NGDAP was 28.5-fold less efficient as a substrate than **L**,**L**-NSDAP.

Hlaváček *et al.*^23^ reported that **L**,**L**-NGDAP did not interact with HiDapE. The disparity between our data and this observation may be a species discrepancy in DapE specificity. However, Hlaváček*et al.*^23^ employed a DapE assay that followed the loss of absorbance due to hydrolysis of **L**,**L**-NSDAP (ε_225 nm_ = 698 M^−1^.cm^−1^), a procedure that is one ninth as sensitive as the NADPH-coupled assay (ε_340 nm_ = 6220 M^−1^.cm^−1^) employed here.

### Response of MtDapE to inhibitors

The assay we developed using our *in situ* substrate synthesis would be of utility for detection of DapE inhibitors which could potentially have antimicrobial properties. Thiols such as **L**-captopril are potent inhibitors of HiDapE^14^ and NmDapE^10^ (K_i_ values 2.8 μM and 1.8 μM respectively). This potency stems partly from co-ordination of the **L**-captopril thiol between the two zinc atoms in the DapE active site^10^. Therefore to extend these studies to MtDapE, we pre-incubated **L**-captopril and the HiDapE thiol-inhibitors **L**-penicillamine^14^ and 2-thiopheneboronic acid^14^ with MtDapE and 31 μM of NS-DAP (the *K*_m_ for this substrate) to determine the impact of these inhibitors on MtDapE.

**L**-Captopril at 10, 3, and 1 mM exerted 99.96%, 26.67% and 23.34% inhibition of MtDapE activity. This inhibition was considerably less than observed for HiDapE^14^ and NmDapE^10^. 1 mM **L**-penicillamine exerted 79.41% inhibition of MtDapE which assuming the competitive kinetics displayed by HiDapE^14^, suggested that the MtDapE K_i_ for this compound would be 28.2 fold greater than that of the HiDapE^14^. 2 thiopheneboronic acid was similarly far less potent an inhibitor of MtDapE (22.3% at 10 mM) than of the HiDapE^14^. Assuming this inhibitor behaved towards MtDapE in a non-competitive manner as it did towards HiDapE^14^, this degree of inhibition suggested MtDapE would be 515-fold less sensitive to 2-thiopheneboronic acid than HiDapE^14^.

The unreactivity of inhibitors such as **L**-captopril towards MtDapE was surprising. We could only detect a single zinc ion in the active site of MtDapE. The structure of the **L**-captopril-inhibited *Neisseria* enzyme revealed the thiol of the inhibitor is sandwiched between two zinc ions^10^ although the loss of one zinc ion does not modify the sensitivity of *Salmonella enterica* DapE to **L**-captopril^15^. It was therefore unlikely that the MtDapE was rendered insensitive to **L**-captopril due to the presence of a single zinc within the active site.

On inspection of the crystal structure of the **L**-captopril complex with NmDapE^10^, N346, G325, Y198 and R179 interact with **L**-captopril. Sequence alignments (ClustalΩ^26^, Supplementary Fig. S4) of the MtDapE with other DapE homologues reveal that these residues are only completely conserved amongst Gram negative organisms. In contrast, actinomycetes including the mycobacteria have substituted NmDapE residues N346, G325, Y198 and R179 with aspartate, tryptophan, arginine and cysteine respectively (Supplementary Fig. S4). These substitutions probably underpin the loss of **L**-captopril potency towards MtDapE^10^.

The insensitivity of mycobacterial DapE to **L**-captopril and other DapE inhibitors underscore the requirement for the development of novel anti-tubercular drugs. Here, we developed a cheap and efficient method to access the **L**,**L**-NSDAP substrate of Mt-DapE that may support future screening for new DapE-directed antimicrobials.

## Methods

### Chemicals, strains and constructs

All the plasmids used in this study are listed in Supplementary Table S1. Restriction endonuclease and other enzymes used for cloning were from New England Biolabs (NEB). Complete EDTA-free protease inhibitor cocktail tablets were from Roche Diagnostics, Germany. Oligonucleotides were from MWG Biotech, Germany. Succinyl-CoA was prepared as in^27^. *meso*-DAP was purified according to^28^. NADP^+^ was from Melford, U.K. All other chemicals used in this study were purchased from Sigma Aldrich. Cellulose TLC plates were from Merck, Darmstadt, Germany.

*E. coli* BL21 (DE3) (Novagen) and C41 (DE3)^29^ was used for expression. The construct of N-terminal His-tagged EcDapD in pFO4^30^ was used in this study. Expression constructs of *Corynebacterium glutamicum meso*-DAP dehydrogenase (Cg*meso*-DAP dehydrogenase) in pET28b^31^ and *Bacillus anthracis* DapF (BaDapF) in pET23a^32^ were kind gifts from Dr. David Roper, University of Warwick, Coventry, U.K.

### Construction of Mt*dapE* and Ec*argD* in pET28b

The Mt*dapE* gene (Rv1202) and *E. coli argD*^33^, (an orthologue of *dapC* in *E. coli* which functions as a *N*-Succinyl-**L**,**L**-DAP aminotransferase) were amplified from *M. tuberculosis* H37Rv genomic DNA and *E. coli* MG 1655 genomic DNA respectively by polymerase chain reaction with Phusion high fidelity DNA polymerase using the primers in Supplementary Table S1. Mt*dapE* and Ec*argD* PCR products were digested with Nde I and Hind III, ligated into similarly digested pET28b vector and transformed into *E. coli* Top10 competent cells. Plasmid DNA was isolated from overnight Luria Bertani (LB) cultures of single transformants grown in the presence of 25 μg/ml kanamycin. The nucleotide sequence of both constructs in frame with a 5′ sequence encoding a (His)_6_ were confirmed by sequencing.

### Over expression of (His)_6_ tagged recombinant proteins

The expression constructs pET28b-MtDapE and pET28b-EcArgD encoding MtDapE and EcArgD were transformed into *E. coli* C41 (DE3). A single transformant was inoculated into a starter culture of LB broth containing 25 μg/ml kanamycin and grown overnight at 37**°**C. A 1% (v/v) inoculum of the starter culture was added per litre of terrific broth (MtDapE) or LB (EcArgD) and cultures were grown in the presence of 25 μg/ml kanamycin at 37°C until an A_600_ of 0.6 was reached. The cultures were then cooled to 16 °C and protein expression was induced with 1 mM isopropyl β-**D**-1-thiogalactopyranoside (IPTG) and incubation was continued at 16 °C for 20 hours. The cultures were harvested and cell pellets were stored at −80 °C.

pFO4-EcDapD showed maximal expression of *E. coli* DapD in BL21 (DE3) cells when induced with 1 mM IPTG at 16 °C for 20 hours. The pET23a-BaDapF and pET28b-Cg*meso*-DAP dehydrogenase constructs were transformed into *E coli* BL21 (DE3) cells and showed maximum expression of Cg*meso*-DAP dehydrogenase and BaDapF when grown for 4 to 5 hours at 37 °C after induction with 1 mM IPTG. Five one litre cultures of BaDapF and two one litre cultures of Cg*meso*-DAP dehydrogenase and EcDapD were grown to obtain sufficient quantities of protein after purification.

### Purification of (His)_6_ tagged recombinant proteins

The cell pellets of MtDapE were resuspended in Buffer A (25 mM N-(2-hydroxyethyl) piperazine-N′-(2-ethanesulphonic acid) (HEPES) pH 7.5, 10% (v/v) glycerol, 50 mM imidazole and 1 mM dithiothreitol (DTT)) supplemented with complete EDTA-free protease inhibitor cocktail and were lysed by sonication at 4 °C with eight 30 seconds pulses interspersed by cooling for 30 seconds. The insoluble pellet was removed by centrifugation at 4 °C and 27,000 × g for 45 minutes. The soluble fraction was purified using a Ni^2+^-loaded HisTrap high performance affinity column (GE Healthcare) which was pre-equilibrated and washed with Buffer A minus the protease inhibitor and eluted isocratically with Buffer A containing 200 mM and then 400 mM imidazole. The purity of the protein was assessed by SDS-PAGE. Fractions containing MtDapE were pooled and dialysed thrice at 4 °C against 2 litres of buffer B (25 mM HEPES pH 7.5, 50% (v/v) glycerol and 1 mM DTT) and the protein was stored in −80 °C.

The same purification protocol was followed for EcArgD and EcDapD except that Buffer A was replaced with Buffer C (20 mM Tris.HCl pH 8.0, 0.5 M NaCl, 10% (v/v) glycerol and 1 mM DTT) and the proteins were eluted with a gradient of 5–500 mM imidazole in Buffer C. Fractions were analysed by 10% SDS-PAGE and the pure proteins were pooled and dialysed three times against 2 litres of Buffer D (20 mM Tris.HCl pH 8.0, 10% (v/v) glycerol and 1 mM DTT). The EcArgD and EcDapD proteins were then concentrated and stored at −80 °C.

BaDapF was purified essentially as described for MtDapE except that Buffer E (50 mM sodium phosphate pH 8.0, 300 mM NaCl, 5 mM imidazole and 1 mM DTT) replaced Buffer A. The column was washed with Buffer E containing 10 mM imidazole followed by buffer E containing 50 mM imidazole and then 250 mM imidazole. The fractions were dialysed against Buffer F (20 mM Tris.HCl pH 8.0, 150 mM NaCl, 5 mM DTT and 50% (v/v) glycerol).

Cg*meso*-DAP dehydrogenase was purified using a HiTrap Q-Sepharose fast flow anion exchange column (GE Healthcare) which was equilibrated and washed with Buffer G (20 mM Tris.HCl pH 8.0 and 1 mM DTT) and eluted isocratically with steps of 350, 400, 450, 500 and 1M NaCl in Buffer G. Fractions containing Cg*meso*-DAP dehydrogenase on SDS PAGE were pooled and dialysed against Buffer H containing 20 mM Tris.HCl pH 8.0, 50% (v/v) glycerol and 1 mM DTT and stored at −80 °C.

### Preparation of metal free-MtDapE and inductively coupled plasma optical emission spectroscopy (ICP-OES) analysis

Metal free MtDapE was prepared by extensive dialysis for three days at 4 °C in Chelex 100-treated 20 mM Tricine buffer pH 8.0 containing 10 mM EDTA and 10 mM 1,10 phenanthroline followed by Chelex 100-treated 20 mM Tricine buffer pH 8.0. The metal content and the metal to protein ratio of MtDapE samples were determined by ICP-OES (Optima 5300 DV, PerkinElmer). Operating conditions were: Air flow rate 13.0 L/min, auxiliary gas flow rate 0.2 L/min, nebuliser flow rate 0.8 L/min and RF power at 1300W. Sulphur (S) and zinc (Zn) contents of the protein were analysed at 180.669 and 181.975 nm for S and 206.200 and 213.857 nm for Zn. MtDapE samples were diluted into 0.1 M ultrapure HNO_3_ and analysed in triplicate, with a washing time of 60 seconds for each sample. Data were analysed using WinLab 32 software (PerkinElmer). The zinc content of the purified MtDapE was calculated by comparison with solutions of known zinc concentrations.

### *In situ* synthesis of L,L-NSDAP and other DAP derivatives

Two 2 mL reactions were set up: Reaction Condition 1 (Schematic in Fig. 1a) contained 20 mM Tricine buffer pH 8.0, 10 mM MgCl_2_, 8 mM *meso*-DAP, 10 mM NADP^+^, 10 mM succinyl CoA, 4.26 μM Cg*meso*-DAP dehydrogenase, 8.03 μM EcDapD, 10 mM **L**-glutamate, 8.97 μM EcArgD, 2 mM pyridoxal phosphate (PLP) and 20 mM DTT. Condition 2, was identical to condition 1, except that the NADP^+^ concentration was reduced to 0.1 mM and in addition contained 950 μg of **L**-glutamate dehydrogenase from *Proteus* species (Sigma Aldrich) and 36.5 mM of ammonium acetate pH 7.6.

The Reaction condition 2 was also used to attempt the synthesis of N-glutaryl-**L**,**L**-DAP (NGDAP), N-acetyl-**L**,**L**-DAP, N-acetoacetyl-**L**,**L**-DAP, N-butyryl-**L**,**L**-DAP, N-malonyl-**L**,**L**-DAP and N-propionyl-**L**,**L**-DAP except that the respective CoA substrates used in the reaction were at 1.25 mM. The reactions were incubated at 37 °C overnight and proteins were removed by centrifugal ultrafiltration through a 10,000 Da cut-off membrane. The ultrafiltrates were separated by anion exchange chromatography using a 40 mL Q Sepharose Source 30Q column (Amersham Biosciences), which was equilibrated with 10 mM ammonium acetate pH 7.6. The samples were diluted 20 fold to 40 ml with 10 mM ammonium acetate pH 7.6 and loaded onto the column, washed with five column volumes of 10 mM ammonium acetate pH 7.6 and eluted with fifteen column volumes of an increasing gradient of 10 to 1000 mM ammonium acetate buffer pH 7.6 at a flow rate of 10 ml/min. Chromatography was followed at 215 nm and fractions were also screened for their respective DAP derivatives by enzymatic assay (below), pooled and lyophilized three times to remove ammonium acetate.

### Electrospray mass spectrometry (ES-MS) and thin layer chromatography (TLC) analysis of L,L-N-acyl-DAP species

Verification of the identity and masses of **L**,**L**-NSDAP and its acyl analogues were sought by negative ion mode ES-MS of a 5 μM sample of either substrate in 50% (v/v) acetonitrile using a Waters G2 Q-TOF mass spectrometer, calibrated with sodium iodide and operating at a capillary voltage of 1.5 kV. The purity of **L**,**L**-NSDAP and **L**,**L**-NGDAP was determined by TLC on cellulose plates developed in methanol:water:concentrated hydrochloric acid:pyridine (90:7.5:2.5:10)^34^, sprayed with 1% (w/v) ninhydrin in ethanol and heated in an oven for a few minutes to develop the purple staining of ninhydrin positive species.

### MtDapE coupled assay

The purified **L**,**L**-NSDAP and derivatives thereof were tested as a substrate in an enzyme assay for MtDapE coupled to DapF. The activity of MtDapE enzyme was measured at 37 °C by following the production of NADPH at 340 nm. Unless indicated otherwise, the standard assay consisted of 20 mM Tricine pH 8.0, 10 mM MgCl_2_, 0.6 mM NADP^+^, 31 μM **L,L**-NSDAP, 1.99 μM Cg*meso*-DAP dehydrogenase, 22.8 μM BaDapF and 0.134 μM MtDapE in a total reaction volume of 200 μl. The reaction was initiated by **L,L**-NSDAP after a 60 to 90 second preincubation of other reaction components and was monitored for 10 minutes. Initial rates were converted to MtDapE activity (min^−1^) assuming a molar extinction coefficient of NADPH of 6220 M^−1^.cm^−1^ at 340 nm.

For characterization of the kinetics of MtDapE substrate utilization, initial rates of MtDapE catalysis were obtained between 10 μM and 320 μM acyl DAP substrate. *K*_m_ and k_cat_ values were extracted from fitting initial rate data to the Michaelis Menten equation with Graphpad prism 5 by non-linear regression. Further characterisation of the temperature and pH optima of the MtDapE enzyme were carried out with the substrate **L**,**L**-NSDAP. To determine the optimum assay temperature for activity the reaction at pH 8.0 was carried out at 25 °C, 30 °C, 37 °C, 42 °C, 45 °C and 50 °C. The impact of pH on activity was examined at 37 °C in the pH range 6.0 to 9.0 with 0.5 unit pH increments at the *K*_m_ of **L**,**L**-NSDAP where the following buffers at 20 mM were used - Sodium acetate (pH 5.5), 2-(*N*-morpholinoethanesulphonic acid (MES) (pH 6), piperazine-N,N′-bis (2-ethanesulphonic acid) (PIPES) (pH 6.5), 3-(*N*-morpholino) propanesulphonic acid (MOPS) (pH 7), HEPES (pH 7.5), Tricine (pH 8) and Tris (hydroxymethyl) aminomethane (Tris.HCl) (pH 8.5 and 9).

### EcDapD coupled assay

EcDapD activity was assayed at 37 °C by generating its **L**-THDP substrate *in situ* and following the production of CoA by its cleavage of dithio*bis*(2-nitrobenzoate) to *para*-thionitrobenzoate at 412 nm^21^. The standard assay consisted of 20 mM Tricine pH 8.0, 10 mM MgCl_2_, 0.3 mM NADP^+^, 0.1 mM 5,5′ dithio*bis*(2-nitrobenzoate), 0.1 mM acyl-Coenzyme A, 0.2 mM *meso*-DAP, 1.99 μM Cg*meso*-DAP dehydrogenase and 10.03 μM EcDapD in a total reaction volume of 200 μl. The reaction was initiated by the addition of EcDapD and continuously monitored. Control assays were performed without *meso*-DAP to take non-enzymatic acyl-CoA hydrolysis into account. Initial rates were converted to EcDapD activity (min^−1^) assuming a molar extinction coefficient of *para*-thionitrobenzoate of 13,600 M^−1^.cm^−1^ at 412 nm.

## Additional Information

**How to cite this article**: Usha, V. *et al.* Reconstruction of diaminopimelic acid biosynthesis allows characterisation of *Mycobacterium tuberculosis N*-succinyl-L,L-diaminopimelic acid desuccinylase. *Sci. Rep.* **6**, 23191; doi: 10.1038/srep23191 (2016).

## Supplementary Material

Supplementary Information

## References

[b1] Dye C. *et al.* WHO and the future of disease control programmes. Lancet. 381, 413–418 (2013). 23374479 10.1016/S0140-6736(12)61812-1

[b2] Kapur A. & Harries A. D. The double burden of diabetes and tuberculosis - Public health implications. Diabetes Res. Clin. Pract. 101, 10–19 (2013). 23305899 10.1016/j.diabres.2012.12.001

[b3] Cox R. J. The DAP pathway to lysine as a target for antimicrobial agents. Nat. Prod. Rep. 13, 29–43 (1996). 8919551 10.1039/np9961300029

[b4] Cox R. J., Sutherland A. & Vederas J. C. Bacterial diaminopimelate metabolism as a target for antibiotic design. Bioorganic Med. Chem. 8, 843–871 (2000). 10.1016/s0968-0896(00)00044-4 10881998

[b5] Schleifer K. H. & Kandler O. Peptidoglycan types of bacterial cell walls and their taxonomic implications. Bacteriol. Rev. 36, 407–477 (1972). 4568761 10.1128/br.36.4.407-477.1972 PMC408328

[b6] Vollmer W., Blanot D. & de Pedro M. A. Peptidoglycan structure and architecture. FEMS. Microbiol. Rev. 32, 149–167 (2008). 18194336 10.1111/j.1574-6976.2007.00094.x

[b7] Scapin G. & Blanchard J. S. Enzymology of bacterial lysine biosynthesis. Adv. Enzymol. Rel. Areas Mol. Biol. 72, 279–324 (1998). 10.1002/9780470123188.ch8 9559056

[b8] Schrumpf B. *et al.* A functionally split pathway for lysine synthesis in *Corynebacterium glutamicum*. J. Bacteriol 173, 4510–4516 (1991). 1906065 10.1128/jb.173.14.4510-4516.1991 PMC208115

[b9] Nocek B. P., Gillner D. M., Fan Y., Holz R. C. & Joachimiak A. Structural basis for catalysis by the mono- and dimetalated forms of the dapE-encoded N-succinyl-l,l-diaminopimelic acid desuccinylase. J. Mol. Biol. 397, 617–626 (2010). 20138056 10.1016/j.jmb.2010.01.062 PMC2885003

[b10] Starus A. *et al.* Inhibition of the dapE-encoded N-succinyl-l,l-diaminopimelic acid desuccinylase from *Neisseria meningitidis* by L-captopril. Biochemistry 54, 4834–4844 (2015). 26186504 10.1021/acs.biochem.5b00475 PMC4671288

[b11] Bienvenue D. L., Gillner D. M., Davis R. S., Bennett B. & Holz R. C. Substrate specificity, metal binding properties, and spectroscopic characterization of the DapE-encoded N-succinyl-l,l-diaminopimelic acid desuccinylase from *Haemophilus influenzae*. Biochemistry 42, 10756–10763 (2003). 12962500 10.1021/bi034845+

[b12] Born T. L., Zheng R. & Blanchard J. S. Hydrolysis of N-succinyl-l,l-diaminopimelic acid by the *Haemophilus influenza dapE* encoded desuccinylase: metal activation, solvent isotope effects, and kinetic mechanism. Biochemistry 37, 10478–10487 (1998). 9671518 10.1021/bi9806807

[b13] Cosper N. J. *et al.* The *dapE*-encoded N-succinyl-l,l-diaminopimelic acid desuccinylase from *Haemophilus influenza* is a dinuclear metallohydrolase. J. Am. Chem. Soc. 125, 14654–14665 (2003). 14640610 10.1021/ja036650v

[b14] Gillner D., Armoush N., Holz R. C. & Becker D. P. Inhibitors of bacterial N-succinyl-l,l-diaminopimelic acid desuccinylase (DapE) and demonstration of *in vitro* antimicrobial activity. Bioorg. Med. Chem. Lett. 19, 6350–6352 (2009). 19822427 10.1016/j.bmcl.2009.09.077

[b15] Uda N. R. *et al.* Zinc-selective inhibition of the promiscuous bacterial amide-hydrolase DapE: implications of metal heterogeneity for evolution and antibiotic drug design. Metallomics 6, 88–95 (2014). 24057071 10.1039/c3mt00125c

[b16] Reinhard L., Mueller-Dieckmann J. & Weiss M. S. Cloning, expression, purification, crystallization and preliminary X-ray diffraction analysis of succinyl-diaminopimelate desuccinylase (Rv1202, DapE) from *Mycobacterium tuberculosis*. Acta. Crystallogr. Sect. F Struct. Biol. Cryst. Commun. 68, 1089–1093 (2012). 10.1107/S174430911203062X PMC3433205 22949202

[b17] Gillner D. M. *et al.* The dapE-encoded N-succinyl-l,l-diaminopimelic acid desuccinylase from *Haemophilus influenza* contains two active-site histidine residues. J. Biol. Inorg. Chem. 14, 1–10 (2009). 18712420 10.1007/s00775-008-0418-z PMC2678232

[b18] Karita M., Etterbeek M. L., Forsyth M. H., Tummuru M. K. & Blaser M. J. Characterization of *Helicobacter pylori dapE* and construction of a conditionally lethal *dapE* mutant. Infect. Immun. 65, 4158–4164 (1997). 9317022 10.1128/iai.65.10.4158-4164.1997 PMC175598

[b19] Pavelka M. S. Jr. & Jacobs W. R. Jr. Biosynthesis of diaminopimelate, the precursor of lysine and a component of peptidoglycan, is an essential function of *Mycobacterium smegmatis*. J. Bacteriol. 178, 6496–6507 (1996). 8932306 10.1128/jb.178.22.6496-6507.1996 PMC178536

[b20] Gillner D. M., Becker D. P. & Holz R. C. Lysine biosynthesis in bacteria: a metallodesuccinylase as a potential antimicrobial target. J. Biol. Inorg. Chem. 18, 155–163 (2012). 23223968 10.1007/s00775-012-0965-1 PMC3862034

[b21] Schuldt L., Weyand S., Kefala G. & Weiss M. S. The three-dimensional structure of a mycobacterial DapD provides insights into DapD diversity and reveals unexpected particulars about the enzymatic mechanism. J. Mol. Biol. 389, 863–879 (2009). 19394346 10.1016/j.jmb.2009.04.046

[b22] Lloyd A. J. *et al.* Characterization of tRNA-dependent peptide bond formation by MurM in the synthesis of *Streptococcus pneumoniae* peptidoglycan. J. Biol. Chem. 283, 6402–6417 (2008). 18077448 10.1074/jbc.M708105200

[b23] Hlaváček J. *et al.* Mono-N-acyl-2,6-diaminopimelic acid derivatives: analysis by electromigration and spectroscopic methods and examination of enzyme inhibitory activity. Analyt. Biochem. 467, 4–13 (2014). 25205653 10.1016/j.ab.2014.08.032

[b24] Vanek V. *et al.* Synthesis of N-succinyl-l,l-diaminopimelic acid mimetics via selective protection. Protein Pept. Lett. 17, 405–409 (2010). 19958280 10.2174/092986610790780387

[b25] Lin Y. K., Myhrman R., Schrag M. L. & Gelb M. H. Bacterial N-succinyl-l,l-diaminopimelic acid desuccinylase. Purification, partial characterisation, and substrate specificity. J. Biol. Chem. 263, 1622–1627 (1988). 3276674

[b26] Sievers F. *et al.* Fast, scalable generation of high-quality protein multiple sequence alignments using clustal omega. Mol. Syst. Biol. 7, 539 (2011). 21988835 10.1038/msb.2011.75 PMC3261699

[b27] Simon E. J. & Shemin D. The preparation of S-succinyl coenzyme A. J. Amer. Chem. Soc. 75, 2520 (1953).

[b28] Koo C. W. & Blanchard J. S. Chemical mechanism of *Haemophilus influenza* diaminopimelate epimerase. Biochemistry 38, 4416–4422 (1999). 10194362 10.1021/bi982911f

[b29] Miroux B. & Walker J. E. Over-production of proteins in *Escherichia coli*: mutant hosts that allow synthesis of some membrane proteins and globular proteins at high levels. J. Mol. Biol. 260, 289–298 (1996). 8757792 10.1006/jmbi.1996.0399

[b30] Nguyen L., Kozlov G. & Gehring K. Structure of *Escherichia coli* tetrahydrodipicolinate N-succinyltransferase reveals the role of a conserved C-terminal helix in cooperative substrate binding. FEBS Lett. 582, 623–626 (2008). 18242192 10.1016/j.febslet.2008.01.032

[b31] Reddy S. G., Scapin G. & Blanchard J. S. Expression, purification and crystallization of meso-diaminopimelate dehydrogenase from *Corynebacterium glutamicum*. Proteins 25, 514–516 (1996). 8865347 10.1002/prot.12

[b32] Matho M. M. An X-ray crystallography challenge: ICAP-1(alpha) and CIB, two integrin-interacting cytoplasmic proteins & structure determination of the reduced form of the diaminopimelate epimerase of *Bacillus anthracis* at 2.4Å resolution. Ph.D. Thesis. Joseph Fourier University, Grenoble, France (2006).

[b33] Ledwidge R. & Blanchard J. S. The dual biosynthetic capability of N-acetylornithine aminotransferase in arginine and lysine biosynthesis. Biochemistry 38, 3019–3024 (1999). 10074354 10.1021/bi982574a

[b34] Kindler S. H. & Gilvarg C. N-succinyl-l,l-2,6-diaminopimelic acid deacylase J. Biol. Chem. 235, 3532–3535 (1960). 13756049

