# Supplementary material for: Reconstruction of diaminopimelic acid biosynthesis allows characterisation of Mycobacterium tuberculosis N-succinyl-L,L-diaminopimelic acid desuccinylase
Source: Sci Rep. 2016 Mar 15;6:23191. doi: 10.1038/srep23191 (PMC4791643; doi:10.1038/srep23191)
Supplement: Supplementary Information [file srep23191-s1.pdf]

Supplementary Information

Reconstruction of diaminopimelic acid biosynthesis allows characterisation of *Mycobacterium tuberculosis N*-succinyl-L,L-diaminopimelic acid desuccinylase

Veeraraghavan Usha[1¶], Adrian J. Lloyd[2¶*], David I. Roper[2], Christopher G. Dowson[2] Guennadi Kozlov[3], Kalle Gehring[3], Smita Chauhan[2], Hasan T. Imam[4], Claudia A. Blindauer[4] and Gurdyal S. Besra[1*]

[1]School of Biosciences, University of Birmingham, Edgbaston, Birmingham B15 2TT, UK, [2]School of Life Sciences, University of Warwick, Coventry, CV4 7AL, UK, [3]Department of Biochemistry, McGill University, Montreal, Quebec, H3G 1Y6, Canada and [4]Department of Chemistry, University of Warwick, Coventry, CV4 7AL,UK.

¶ These authors contributed equally to this work.

*Corresponding author e-mail: g.besra@bham.ac.uk and Adrian.Lloyd@warwick.ac.uk

Supplementary Figure Legends

Figure S1. Expression and purification of MtDapE, Cg*meso*DAP dehydrogenase, BaDapF, EcArgD and EcDapD. SDS-PAGE analysis of purification of MtDapE, Cg*meso*DAP dehydrogenase, BaDapF, EcArgD and EcDapD. Cg*meso*DAP dehydrogenase was purified by anion exchange on HiTrap Q-Sepharose from which it was eluted isocratically with NaCl, whilst all other proteins were purified on Ni^{2+} loaded His Trap high performance affinity columns from which they were eluted isocratically with imidazole. **(a) MtDapE** – Lanes 1-4 respectively: 50 mM imidazole wash, 200 mM imidazole eluate, 400 mM imidazole eluate and molecular weight markers. **(b) Cg*meso*Dap dehydrogenase** – Lanes 1: molecular weight markers, lanes 2-6 respectively: elution with 350, 400, 450, 500 and 1M NaCl. **(C) BaDapF** – Lanes 1-3 respectively: crude extract, 10 mM imidazole wash and 50 mM imidazole wash. Lanes 5-8. 250 mM imidazole washes. **(d) EcArgD** – Lane 2: crude extract, lane 3: molecular weight markers, lane 4: flow through, lanes 5-12 respectively: 10, 25, 50, 100, 200, 300, 400 and 500 mM imidazole washes. **(e) EcDapD** – Lane 1: crude extract, lane 2: overspill of sample from lane 1, lanes 3-7 respectively: 5, 10, 25, 50 and 100 mM imidazole washes, lane 12: molecular weight markers.

Figure S2. Assay of EcDapD succinylation of L-THDP generated *in situ* by Cg*meso*-DAP dehydrogenase. (a) Flow diagram of the EcDapD assay: *meso*-DAP is oxidised by Cg*meso*-Dap dehydrogenase to **L**-THDP which is then succinylated with succinyl-CoA (or acylated by an alternative acyl-CoA) by EcDapD to yield NS-AKP and CoA. The CoA thiol reduces the chromogen disulphide 5,5'dithio*bis* (2-nitrobenzoic acid) to a mixed disulphide and the *para*-thionitrobenzoate chromophore which absorbs at 412 nm. **(b) Assay of EcDapD activity:** Conditions are described in methods section. Sufficient Cg*meso*-Dap

dehydrogenase and $NADP^+$ was added to assure complete conversion of the *meso*-DAP to **L**-THDP prior to addition of 5,5'dithio*bis* (2-nitrobenzoic acid) to the assay. Blue trace: assay carried out in the presence of 0.14 mM *meso*-DAP, green trace: assay carried out omitting the *meso*-DAP.

Figure S3. Temperature and pH dependence of MtDapE activity. All assays were carried out at 0.134 ∝M MtDapE and 31 ∝M **L**,**L**-NSDAP. All data points are means of duplicates that differ by no more than 10%. **(a) The effect of temperature on MtDapE catalysis**: This was determined between 25 and 50°C. **(b)-(d): The effect of pH on MtDapE activity: (b)** The pH profile for MtDapE activity was determined between pH 6 to 9 with 0.5 increments. Assays were carried out using standard concentrations of coupling enzymes (1.99 ∝M Cg*meso*-DAP dehydrogenase and 22.8 ∝M BaDapF). To establish the coupling enzymes were not limiting the MtDapE rate, the experiment was repeated where the Cg*meso*-DAP dehydrogenase concentration was increased four-fold to 7.96 ∝M **(c)** and where the BaDapF concentration was increased four-fold to 91.2 ∝M **(d).**

Figure S4. ClustalΩalignment of sequences of DapE highlighting residues involved in zinc or L-captopril binding. Clustal Ω alignment of sequences of DapE obtained from the National Center of Biotechnology Information (http://www.ncbi.nlm.nih.gov/protein/?term=DapE). Accession numbers were: *M. tuberculosis*YP_177796.1, *M. leprae*CAC31440.1, *M. smegmatis*AFP41419.1, *C. diptheriae*CAE49500.1, *Streptomyces coelicolor*NP_629287.1, *N. meningitidis* NP_274537.1, *H. influenza* NP_438276.1, *E. coli*CAA40665.1, *Staphylococcus aureus*CEK45041.1, *Streptococcus pneumoniae*CKF71638.1, *Pseudomonas aeruginosa*KFL13351.1, *Acinetobacter baumanii*ADX04743.1, *Burkholderia*

malleiALC56873.1. Sequences were aligned according to Clustal Ω default settings. Numbering above the *M. tuberculosis* sequence corresponds to the MtDapE sequence. Zinc binding residues (identified in the HiDapE and NmDapE crystal structures) are red, highlighted in yellow. Positions of residues in the sequence alignment implicated in **L**-captopril binding in the HiDapE and NmDapE crystal structures are indicated by the letters Cap (white on black background). Conserved residues (or conservative substitutions thereof) implicated in **L**-captopril binding from the NmDapE crystal structure are white numbers highlighted in black. Non-conservative substitutions of these residues are white numbers highlighted in grey. The actinomycete sequences are highlighted in purple.

Supplementary Reference

S1. Nguyen, L., Kozlov, G. & Gehring, K. Structure of *Escherichia coli* tetrahydrodipicolinate N-succinyltransferase reveals the role of a conserved C-terminal helix in cooperative substrate binding. *FEBS. Lett.* **582,** 623-626 (2008).

Supplementary Figure S1

Expression and purification of MtDapE, Cg*meso*DAP dehydrogenase, BaDapF, EcArgD and EcDapD

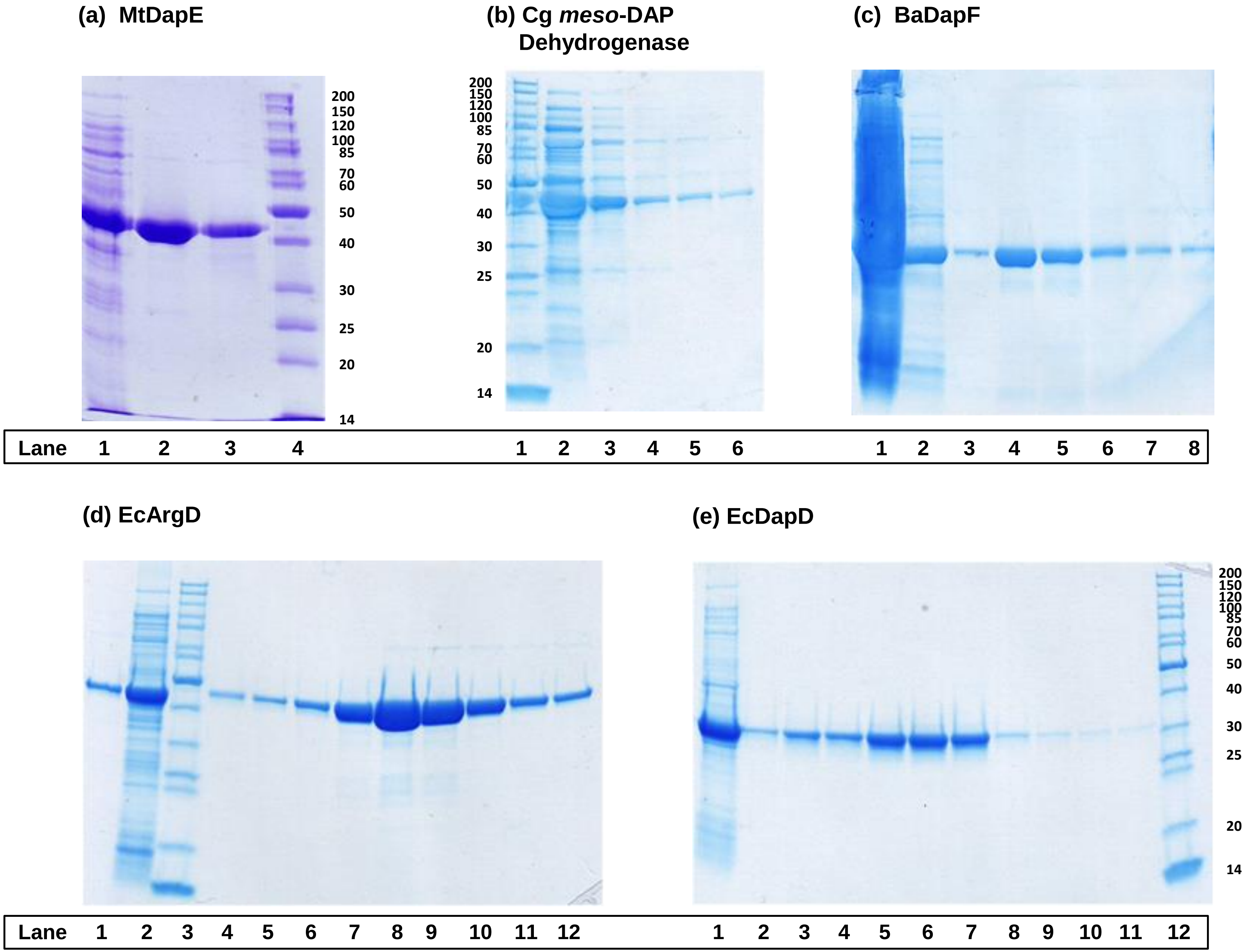

Supplementary Figure S2

Assay of EcDapD succinylation of L-THDP *in situ* by Cg*meso*-DAP dehydrogenase

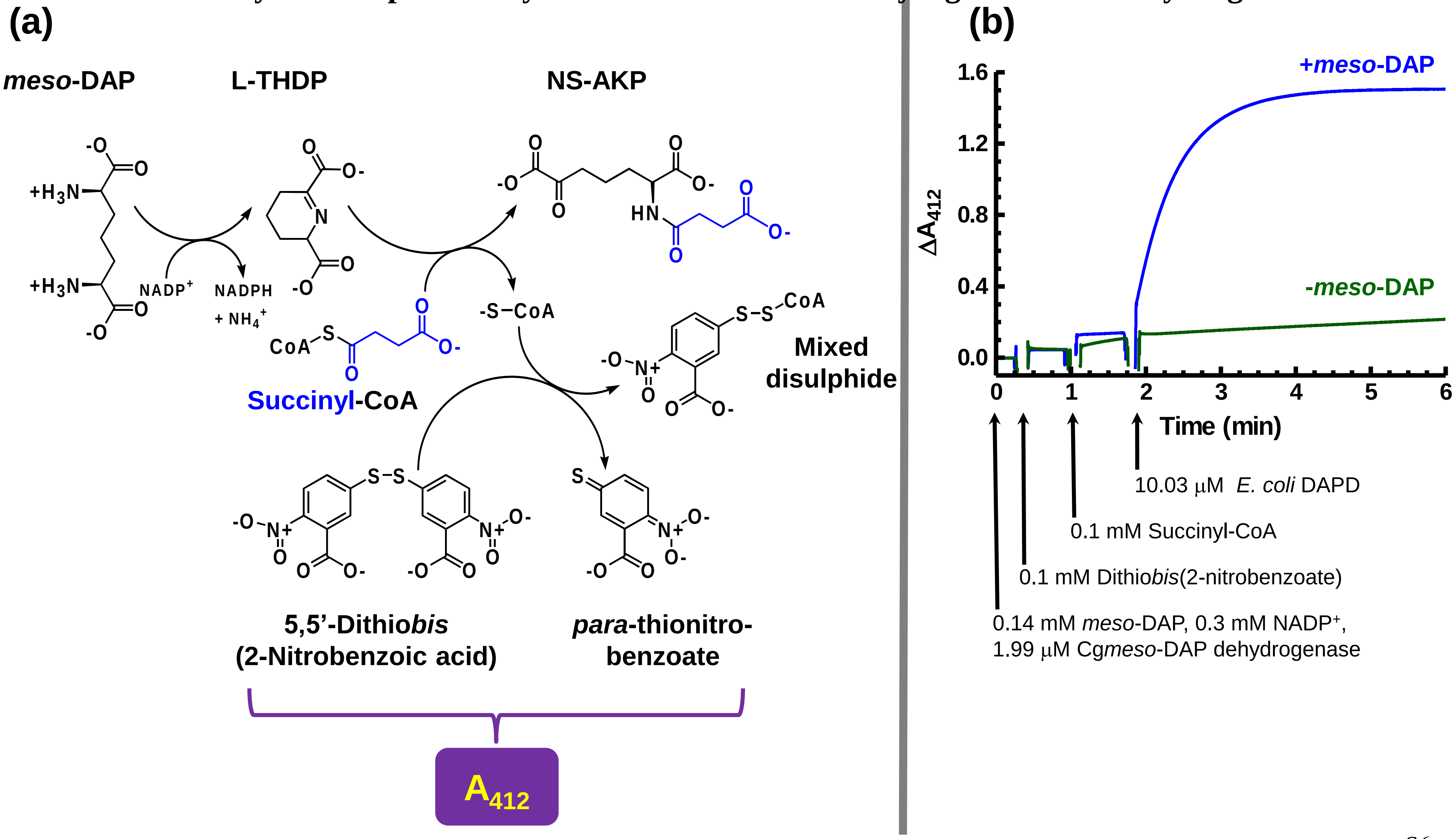

Supplementary Figure S3

Temperature and pH dependence of MtDapE activity

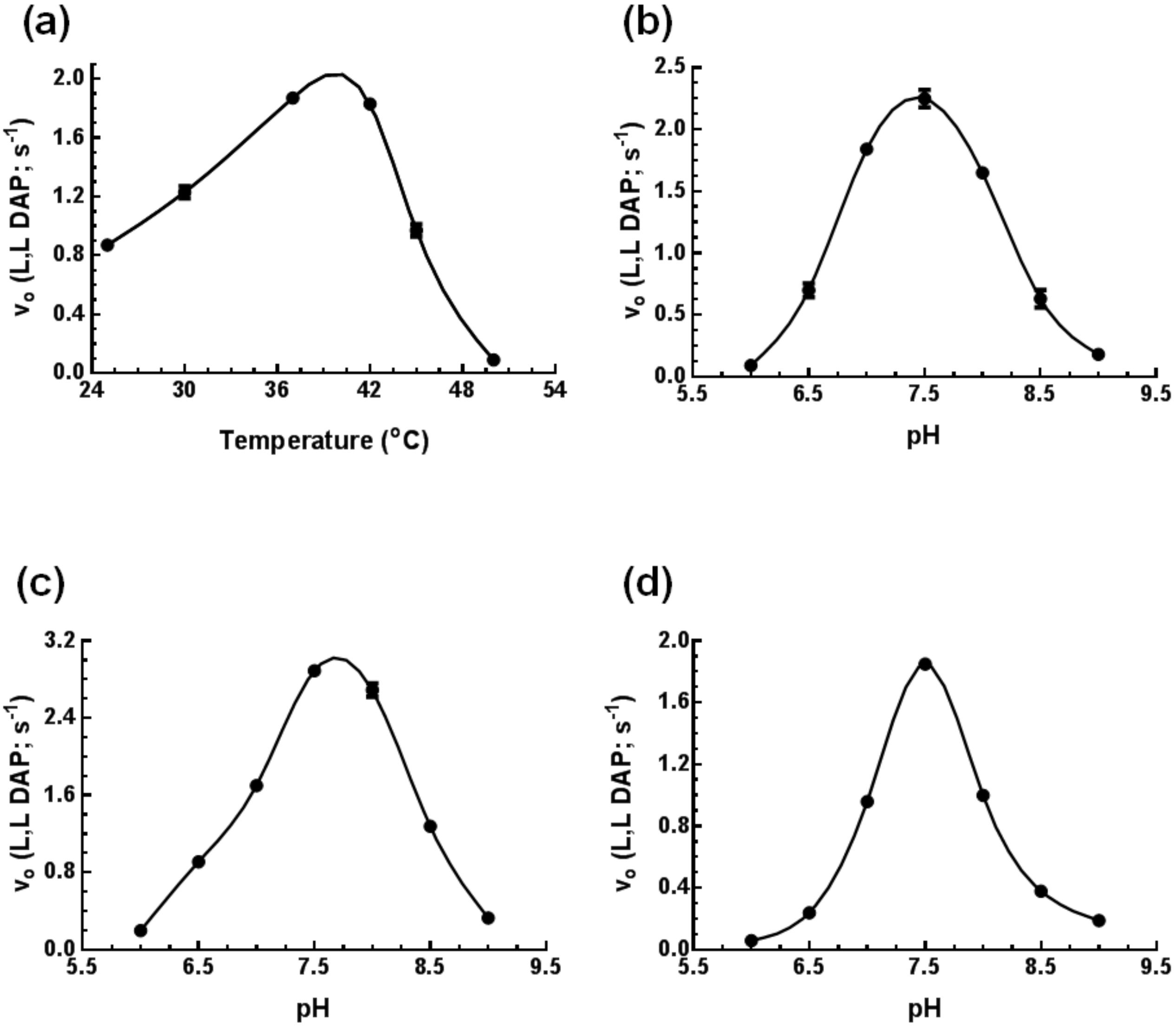

Supplementary Figure S4

Clustal Ω Alignment of sequences of DapE highlighting residues involved in zinc or L-captopril binding

```
                                                                                                                 68                                       95                                        126
Mycobacterium tuberculosis    ------M----------LDLRGDPIELTAALIDIPSESRK---EARIADEVEAALRAQASGFEIIRNGNA---VLAR--TKLNRSSRVLLAGHLDTVPVAGNL-----P--SRRE-NDQLHGCGAADMKSGDAVFLHLAA-TLA---EPTHDLTLVFYDCEEIDSAANGLGRI---QREL  141
Mycobacterium leprae          ------M----------LDLRGDPIELTAALVDIPSESRA---EARIADAVETALRAQTAGFEIVRNGNA---VLAR--TSLHRPARVLLAGHLDTVPVAGNL-----P--SRCE-KDVLHGCGTVDMKSGDAVFLHLAA-TVT---EPVHDLTLMFYDCEEIDSAANGLVRI---EREL  141
Mycobacterium smegmatis       -----MG----------LDLSADPIALTAALVDIPSVSRD---ERRIADEIEAALRAQAPHFEVIRNGDA---VLAR--TNLGRPSRVMLAGHIDTVPIADNL-----P--SRIV-DGEMYGCGTSDMKAGDAVFLHLAA-TIT---EPTHDITLVMYDCEEIESSANGLGRI---EREL  142
Corynebacterium diptheriae    ------M----------LDLTTNPIDLTRALVDIESPSHY---EKNIADAIEQALHA-IDNVEVLRHGNT---LVAR--THRGLGERVILAGHIDTVPIADNV-----P--SHME-GDVMFGCGTVDMKSGMAVYLHAFA-TLANHPALAKDLTVVCYEGEEVASEYNGLGHV---QAAH  143
Streptomyces coelicolor       --MADTP----------LDLTLDAAELTARLVDFPSESGT---EKPLADAIESALRT-LPHLSVERYGNN---LVAR--TGLGRAERVILAGHIDTVPIAGNV-----P--SRLDEDGVLWGCGTCDMKAGVAVQLRIAA-TVP---APNRDLTFVFYDNEEVAAELNGLKHV---AEAH  145
Neisseria meningitidis        -----MTETR------------SLELAKELIARPSVTPD---DQNCQKLLAERLHKIGFAAEELHFGDTKNIWLRR----GTKVPVVCFAGHTDVVPTGPVEKWDSPPFEPTERD-GRLYGRGAADMKTSIACFVTACERFVAEHPDHQGSIALLITSDEEGDALDGTTKVVDVLKARG  154
Haemophilus influenzae        ------MKEK------------VVSLAQDLIRRPSISPN---DEGCQQIIAERLEKLGFQIEWMPFNDTLNLWAKH----GTSEPVIAFAGHTDVVPTGDENQWSSPPFSAEIID-GMLYGRGAADMKGSLAAMIVAAEEYVKANPNHKGTIALLITSDEEATAKDGTIHVVETLMARD  153
Escherichia coli              ------MSCP------------VIELTQQLIRRPSLSPD---DAGCQALLIERLQAIGFTVERMDFADTQNFWAWR----GQGE-TLAFAGHTDVVPPGDADRWINPPFEPTIRD-GMLFGRGAADMKGSLAAMVVAAERFVAQHPNHTGRLAFLITSDEEASAHNGTVKVVEALMARN  152
Staphylococcus aureus         --MTTFSEKE------------KIQLLADIVELQTENNN---EIDVCNYLTDLFDKYDIKSEILKVNEH---RANIVAEIGNGSPILALSGHMDVVDAGNQDNWTYPPFQLTEKA-GKLYGRGTTDMKGGLMALVITLIELKEQNQLPQGTIRLLATAGEEKEQEGAKLLAD---KGYL  155
Streptococcus pneumoniae      --MVFPSEQEQIEKFEKDHVVQHYFEVLRTLISKKSVFAQQVGLKEVANYLGEIFKRVGAEVEIDESYTAP-FVMAHFKSSRPDAKTLIFYNHYDTVPADGDQVWTEDPFTLSVRN-GFMYGRGVDDDKGHITARLSALRKYMQRHDDLPVNISFIMEGAEESASTDLDKY---LEKHAD  172
Pseudomonas aeruginosa        MTASSPSLSP------------TLELACELIRRPSVTPL---DADCQALMMRRLEAAGFALEPMRIEEVDNFWARR----GGDGPVLCFAGHTDVVPTGPLQAWQHQPFDALIDDQGMLCGRGAADMKGSLASMIVAVERFVADHPEHKGAIAFLITSDEEGPAHHGTKAVVERLAARG  160
Acinetobacter baumanii        -----MNHSD------------TLSLSLELLQQPSVTPI---DHTCQTIIAERLAKVGFHIEPMRFGDVDNLWARR----GTEGPVFCFAGHTDVVPTGRLDAWNSDPFAPEIRD-GKLYGRGSADMKTALAAMVVASERFIAKHPNHKGSIAFLITSDEEGPAVNGTVKVIETLEKRN  154
Burkholderia pseudomallei     -------MSA------------TLALTEQLIARASVTPD---DQHCQQLMIERLAALGFECETIASHGVTNFWAVKRGTAGRAGKLLAFAGHTDVVPTGPLEQWSSPPFVPTHRD-GKLYGRGAADMKTSLAGFVVAAEEFVAAHPQHRGSIGFLITSDEEGPATDGTVKVVEALAARG  156
```

```
                                          154155       164                183
Mycobacterium tuberculosis    PDWLSADVAILGEPTA-----GCIEAGCQGTLR--VVLSVTGTRAHSARSWLGDNAIHKLGAVLDRLAVYRARS---------------------------------------------VDIDGCTYRE-GLSAVR----VAGGVAGNVIPDAASVTINYRFAPDRSVAAALQHVHDVF  263
Mycobacterium leprae          PEWLAADVAILGEPTG-----GYIEAGCQGTLR--VVINAAGSRAHSARSWLGDNAIHKLGAVLDRLAAYRART---------------------------------------------VDIDGCVYRE-GLSAVR----VDGGVAGNVIPDAASVTVNYRFAPDRSVAAALRHVHDVF  263
Mycobacterium smegmatis       RDWLTADVAILGEPSG-----GYIEAGCQGTIR--VVISAKGTRAHSARSWLGDNAIHKLAPVLDRLASYQARS---------------------------------------------VDIDGCVYRE-GLSAVR----IDGGIAGNVIPDAASVTVNFRFAPDRDVDQAVAHVHEVF  264
Corynebacterium diptheriae    PDWLSGDLALLGEPSG-----AVIEAGCQGTIR--LRVTAHGVRAHSARSWLGDNAMHKLAPVITNIAQYHAQE---------------------------------------------MLVDGCLYHE-GLNIVH----CESGVATNTIPDEAWMFVNFRFAPNRSVDEALAHMLEVL  265
Streptomyces coelicolor       PEWLEGDFAVLLEPSD-----GQVEGGCQGTLR--VLLKTAGERAHSARSWMGSNAIHAAAPILARLAAYEPRY---------------------------------------------PVIDGLEYRE-GLNAVG----ITGGVAGNVIPDECVVTVNFRYAPDRSPEEALAHVREVF  267
Neisseria meningitidis        ELI---DYCIVGEPTAVGKLGDMIKNGRRGSLS--GNLTVKGKQGHIAYPHLAINPVHTFAPALLELT-------------------------------------------------QEVWDEGNEYFPPTSFQISNI---NGGTGATNVIPGELNVKFNFRFSTESTEAGLKQRVHAIL  277
Haemophilus influenzae        EKI---TYCMVGEPSSAKNLGDVVKNGRRGSIT--GNLYIQGIQGHVAYPHLAENPIHKAALFLQELT-------------------------------------------------TYQWDKGNEFFPPTSLQIANI---HAGTGSNNVIPAELYIQFNLRYCTEVTDEIIKQKVAEML  276
Escherichia coli              ERL---DYCLVGEPSSIEVVGDVVKNGRRGSLT--CNLTIHGVQGHVAYPHLADNPVHRAAPFLNELV-------------------------------------------------AIEWDQGNEFFPATSMQIANI---QAGTGSNNVIPGELFVQFNFRFSTELTDEMIKAQVLALL  275
Staphylococcus aureus         DDV---DGLIIAEPTG----SGIYYAHKGSMS---CKVTATGKAVHSSVPFIGDNAIDTLLEFYNQFKEKY---------SELKKHD----------TKHELDVAPMFKSLI----GKEISEEDANYASGLTAVCSI---INGGKQFNSVPDEASLEFNVRPVPEYDNDFIESFFQNII  298
Streptococcus pneumoniae      KLRGADLLVWEQGTKNALEQLEISGGNKGIVTFD-AKVKSADVDIHSSYGGVVESAPWYLLQALQSLRAADGRILVEGLYEEVQEPNEREMALLETYGQRNPEEVSRIYGLELPLLQEDRMAFLKRFFFEPALNIEGIQSGYQGQGVKTILPAEASAKLEVRLVPGLEPHDVLEKIRKQL  352
Pseudomonas aeruginosa        ERL---DWCIVGEPSSTSLVGDVVKNGRRGSLG--AKLTIRGVQGHVAYPHLAKNPIHLAAPALAELA-------------------------------------------------AEHWDDGNAFFPPTSFQVSNL---NSGTGATNVIPGELTALFNFRFSTESTVEGLQKRVEAIL  283
Acinetobacter baumanii        EKI---TWCLVGEPSSTHKLGDIVKNGRRGSLN--AVLKVQGKQGHVAYPHLARNPIHEASPALAELC-------------------------------------------------QTVWDNGNEYFPATSFQISNI---HAGTGATNVIPGALEVTFNFRYSTEVTAEQLKQRVHEIL  277
Burkholderia pseudomallei     ERL---DYCIVGEPTSTATLGDVVKNGRRGSMS--GELVVKGVQGHIAYPHLAKNPIHLLAPALAELA-------------------------------------------------AEQWDEGNEYFPPTTWQVSNL---RAGTGATNVIPGHADLLFNFRFSTASTVEGLQARVHAIL  279
                                           Cap         Cap                Cap
```

```
                                                                                      305                  325    330
Mycobacterium tuberculosis    DGLDVQIEQ---TDAAAGALPGLSEPAAKALVEAAGG-----------QVRAKYGWTDVSRF-AAL--GIPAVNYGPGDPNL-AHCRDERVPVGNITAAVD----LLRRYLGG--------  354
Mycobacterium leprae          DGLDVQIEQ---TDAAVGALPGLSQPAAKALVEAADG-----------QVRAKYGWTDVSRF-AAL--GVPAVNYGPGDPNL-AHRHDERVPVVQITAAVD----MLRRYLGG--------  354
Mycobacterium smegmatis       DGLDVTLEL---TDAAAGALPGLTQPAAAALVAAAGG-----------QVRAKYGWTDVARF-AAL--GIPAVNYGPGDPNL-AHRVDERVQVAAITAAAD----MLRTYLTT--------  355
Corynebacterium diptheriae    DLPEGVTYDI--DDAVPGARPGLDRPAAAALLEATGG-----------KFRAKYGWTDVSRF-SEI--GVPAVNFGPGDPAY-CHKKDEQCPVSMITEVSR----TLVHYLTSTNSQTTGA  365
Streptomyces coelicolor       DGCGVTDFVV--DDHSGAALPGLSHPAAAAFIEAVGG-----------TPQPKYGWTDVSRF-SAL--GVPAVNYGPGNPHL-AHKRDERVEVAKILAGEE----RLRSWLTA--------  359
Neisseria meningitidis        DK---HGVQY-DLQWSCSGQPFLTHA-GK-LTDVARAAIAEICGVET-EL-STTGGTSDGRFIKAI--AKELIELGSSNAT--IHQINENVRLDDIPKLSAVYEGILARLLAGNAV-----  381
Haemophilus influenzae        EK---HNLKY-RIEWNLSGKPFLTKP-GK-LLDSITSAIEETIGITP-KA-ETGGGTSDGRFIALM--GAEVVEFGPLNST--IHKVNECVSVEDLGKCGEIYHKMLVNLLDS--------  377
Escherichia coli              EK---HQLRY-TVDWWLSGQPFLTAR-GK-LVDAVVNAVEHYNEIKP-QL-LTTGGTSDGRFIARM--GAQVVELGPVNAT--IHKINECVNAADLQLLARMYQRIMEQLVA---------  375
Staphylococcus aureus         NDVDSNKLSL-DI--PSNHRPVTSDKNSK-LITTIKDVASSYVEQDDIFVSALVGATDASSFLGDNKDNVDLAIFGPGNPLM-AHQIDEYIEKDMYLKYIDIFKEASIQYLKEK-------  407
Streptococcus pneumoniae      DK---NGFDKVELYYTLGEMSYRSDMSAPAILNVIELAKKFY-PQGVSVLPTTAGTGPMHTVFDAL--EVPMVAFGLGNANSRDHGGDENVRIADYYTHIELVEELIRSYE----------  457
Pseudomonas aeruginosa        DK---HGLDW-HVEWALSGLPFLTEP-GE-LLDAVAASIRAVTGRET-QP-STSGGTSDGRFIATM--GTQVVELGPVNAT--IHQVNERVLASDLELLTEIYYQTLVRLLA---------  383
Acinetobacter baumanii        DK---HGLQY-EIVWNLSGLPFLTPV-GE-LVNAAQTAILNVTGTET-EL-STSGGTSDGRFIAPT--GAQVLELGVLNAT--IHQINEHVDVHDLDPLTDIYEQILENLLAQ--------  378
Burkholderia pseudomallei     DR---HGLDY-TLNWSVSGLPFLTPR-GE-LSNALDAAIRAETGVSP-EL-STTGGTSDGRFIARI--CPQVIEFGPPNAS--IHKIDEHIDVRFVDPLKNVYRRVLEQLIA---------  379
                                                                                      Cap                  Cap
```

330: Residues involved in zinc binding (*M. tuberculosis* sequence numbering)

Cap: Conserved residues involved in **L**-captopril binding in DapE species sensitive to the drug

325:Non-conservativesubstitutions of residues involved in **L**-captopril binding

Supplementary Table S1. Plasmids and PCR primers used in this study.

Plasmid or PCR primer	Description of plasmid or Sequence 5'-3'.	Source or Reference
pFO4/EcDapD	pFO_4 containing full length *dapD* gene of *E.coli* for overexpression, ampicillin resistance.	Nguyen *et. al.*[S1].
pET23a/BaDapF	pET23a containing *dapF* of *B. anthracis*. ampicillin resistance	Dr. David Roper Warwick University
pET28b/Cg*meso*-DAP dehydrogenase	pET28b containing the *dapdehydrogenase* gene of *C. glutamicum*. kanamycin resistance	Dr. David Roper Warwick University
pET28b/EcArgD	pET28b containing full length *argD* of *E. coli*. kanamycin resistance	This study
Ec*argD* Forward primer	ta**catatg**gcaattgaacaaacagcaattac	This study
Ec*argD* Reverse primer	gc**aagctt**ttacgccccaaccaccttc	This study
pET28b/MtDapE	pET28b containing full length *dapE* of *M. tuberculosis* H37Rv. kanamycin resistance	This study
Mt*dapE* Forward primer	gatcgatc**catatg**ctggatttgcgcggggac	This study
Mt*dapE* Reverse primer	gatcgatc**aagctt**ctagccacccaggtatcgg	This study
